# The Clinical and Genetic Features of Co-occurring Epilepsy and Autism Spectrum Disorder in Chinese Children

**DOI:** 10.3389/fneur.2019.00505

**Published:** 2019-05-14

**Authors:** Shasha Long, Hao Zhou, Shuang Li, Tianqi Wang, Yu Ma, Chunpei Li, Yuanfeng Zhou, Shuizhen Zhou, Bingbing Wu, Yi Wang

**Affiliations:** ^1^Department of Neurology, Epilepsy Center, Children's Hospital of Fudan University, Shanghai, China; ^2^Department of Neurology, Guizhou Provincial People's Hospital, Medical College of Guizhou University, Guizhou, China; ^3^Key Laboratory of Birth Defects, Children's Hospital of Fudan University, Shanghai, China

**Keywords:** epilepsy, ASD, whole exome sequencing, copy number variants, voltage-gated ion channel gene, epilepsy syndrome

## Abstract

There is still no comprehensive description of the general population regarding clinical features and genetic etiology for co-occurring epilepsy and autism spectrum disorder (ASD) in Chinese children. This study was a retrospective study of children diagnosed with epilepsy and ASD from January 1st, 2015, to May 1st, 2018, at the Children's Hospital of Fudan University. A total of 117 patients met the inclusion criteria, and 103 subjects were eligible. Among them, 88 underwent genetic testing, and 47 children (53.4%) were identified as having pathogenic or likely pathogenic variants: 39 had single gene mutations (83.0%, 39/47), and eight had copy number variants (17.0%, 8/47), with *SCN1A* (14.9%, 7/47) and *MECP2* (10.6%, 5/47) gene mutations being the most common. Mutations in other genes encoding voltage-gated ion channels including *SCN2A, CACNA1A, CACNA1H, CACNA1D*, and *KCNQ2* were also common, but the number of individual cases for each gene was small. Epilepsy syndrome and epilepsy-associated syndrome were more common (*P* = 0.014), and higher rates of poly-therapy (*P* = 0.01) were used in the positive genetic test group than in the negative group. There were no statistically significant differences in drug-refractory epilepsy, ASD severity, or intellectual disability between the positive genetic test group and the negative genetic group. These data strongly indicate the need for ASD screening in children with epilepsy with voltage-gated ion channel gene variants for better diagnosis and early intervention.

## Introduction

Epilepsy, especially drug-refractory epilepsy, with ASD is a chronic neurologic disorder but also places a substantial burden on children, families and society (1). Previous research reported that the comorbidity rate varied widely, even in large samples including population-based studies, ranging from 2.4 to 63% (2, 3). In different epilepsy syndromes, such as in tuberous sclerosis complex (TSC), the incidence of ASD is 17–63% (4). In Dravet syndrome (DS), the incidence of ASD is 24–61% (5, 6), and in infantile spasms, it is 35% (7). Most published studies have primarily focused on clinical characteristics and outcomes of epilepsy with ASD (8). However, to date, there is still no comprehensive general description of both clinical features and the genetic etiology of these disorders.

In clinical work, we found large heterogeneity in behaviors and outcomes in epilepsy with ASD children, and the reasons are still unclear. In recent years, with whole exome sequencing (WES), neural panel data sequencing, copy number variant (CNV) analysis, and other molecular technologies, increasing numbers of epilepsy with ASD-related diseases have been recognized at the molecular level, such as Angelman syndrome, TSC, Fragile X syndrome, and Rett syndrome. To date, a small number of gene mutations, such as in *SCN1A* and *TSC2*, are known to cause epilepsy and ASD phenotypes simultaneously. Recently, several reviews described epilepsy with ASD genes from the perspectives of bioinformatics and genetics (9–11). However, approximately 50% or more of the causes remain unknown. We therefore sought to expand the candidate genes for epilepsy with ASD and determine whether genetically pathogenic patients are more prone to drug-refractory epilepsy, severe ASD, and intellectual disability than non-pathogenic patients. Thus, in the current study, we conducted genetic testing on children with co-occurring epilepsy and ASD to identify causative genetic variants to determine a possible link between genotypes and phenotypes.

## Participants and Methods

### Study Design

Children with epilepsy and ASD were retrospectively recruited from January 1st, 2015, to May 1st, 2018, at the National Children's Medical Center, Shanghai, China. Primary data were collected from electronic clinical history systems and medical records from both inpatients and outpatients at the Children's Hospital of Fudan University. The key words “autism or ASD and epilepsy or seizure” were used to search the clinical database for patients.

### Definitions and Measurements

1. Epilepsy:
  (1) Epilepsy was diagnosed based on the International Classification of Diseases−10. Seizure type and epilepsy syndrome were defined according to the criteria of the Commission on Classification and Terminology of the International League Against Epilepsy in 2010. Epilepsy-associated syndrome is considered a well-defined syndrome with an epileptic phenotype in published literature but not a defined epilepsy syndrome by the International League Against Epilepsy.
  (2) Drug-refractory epilepsy was defined as failure with adequate trials of at least two tolerated, appropriately chosen anti-epileptic schedules to achieve a sustained seizure-free state for <1 year (12).
2. ASD was assessed with the Autism Behavior Checklist, completed by parents, for patients at least 18-month-old with core symptoms of ASD. When the score on the questionnaire was >61, ASD was considered. Then, all ASD patients were diagnosed by qualified and experienced neurologists and psychologists using the Diagnostic and Statistical Manual of Mental Disorders V (DSM-V) published in 2013 and based on the patient's clinical behavior. Clinical judgment was based on all available questionnaires including the ADOS (Autism diagnostic observation schedule) and the ADI-R (Autism diagnostic interview-revised). ASD severity was assessed using the DSM-V, which was divided into mild, moderate, or severe (13).
3. Intellectual disability (ID) was defined as an IQ lower than 70 with accompanying adaptive behavior deficits. Mild ID was defined as 50 < IQ ≤ 70, and moderate to severe ID was defined as an IQ < 50. IQ was acquired using development screening testing, and some of the children took the Wechsler Intelligence Scale.
4. Brain dysplasia on magnetic resonance imaging (MRI) included hypoplasia in the cerebellar hemispheres and vermis, dysmorphometry of the left and right olfactory bulbs and sulci, abnormalities in the hippocampus and choroid plexus, and malformation of the corpus callosum (14). The malformations in cortical development were assessed using Barkovich criteria (15).

### Inclusion Criteria

(1) All children aged 1–18 years diagnosed with epilepsy with ASD from January 1st, 2015, to May 1st, 2018, and (2) all subjects followed up at least 1 year after diagnosis and treated with anti-epileptic drugs (AEDs) were included.

### Exclusion Criteria

Children with (1) acute symptomatic epilepsy, including head trauma, brain tumors, and infections of the central nervous system, (2) ASD-like behaviors but failing to meet with DSM-V criteria, or (3) incomplete clinical information or failure to follow-up were excluded.

### Ethical Approval

The study was approved by the Children's Hospital of Fudan University ethics committee (NM: (2016) 117). All participants or their parents were made aware of the research and signed written consent forms.

### Study Participants and Data Collection

All patient medical records were collected, including sex, onset age of seizures and ASD symptoms, seizure types, AEDs used, electroencephalogram (EEG) including sleeping EEG and video EEG, head MRI, family history, perinatal abnormalities, genetic tests, and other neuropsychiatric comorbidities. If data were incomplete, further information was obtained by telephone interview.

### Genetic Testing

Pathogenic copy-number variants were tested using array-based comparative genomic hybridization. Next generation sequencing was used with a targeted neuromuscular gene panel including 2,732 genes or WES. Target ranges were enriched by Agilent (Santa Clara, CA, USA) ClearSeq Inherited Disease panel kits or Agilent SureSelectXT Human All Exon 50-Mb kits and sequenced with an Illumina Hiseq platform. The average sequencing depth of the target region was 100–200×, and the 98% depth of the target sequence reached 20× or more. Both the neuromuscular panel and whole exome sequencing can detect more than 1 Mb copy number abnormalities. If a copy number anomaly was found during the analysis, it was indicated in the report.

Gene variant annotations were referred to using American College of Medical Genetics and Genomics (ACMG) guidelines. According to ACMG guidelines, a variant is considered to be pathogenic when (1) this variant strongly explains the indication for testing and may be responsible for patient phenotype, or (2) the same nucleotide and amino acid change has been reported previously as a pathogenic variant or existed in internal database. A likely pathogenic variant is considered when (1) the variant likely explains the indication for testing and may be responsible for patient phenotype, (2) a *de novo* variant (parent sanger sequencing confirmed) is found in the proband without family history or inherited from the affected parents, (3) regardless of nucleotide change, amino acid change is the same as a previously pathogenic variant, and (4) the null variant, includined g non-sense, frameshift, canonical ± 1 or 2 splice sites, initiation codon, single, or multiexon deletion in a gene, where is considered loss of function According to previous literature (16). Positive results need to follow Mendel's law of inheritance: (1) an autosomal dominant or X-linked dominant gene mutation includinged one heterozygous pathogenic or likely pathogenic variant, (2) an autosomal recessive gene mutation includinged one homozygous or two heterozygous pathogenic or likely pathogenic variants (compound heterozygous), or (3) X-linked recessive genes in male patients including one pathogenic or likely pathogenic variant. All variants were searched in OMIM (https://www.ncbi.nlm.nih.gov/omim/) and HGMD (http://www.hgmd.cf.ac.uk/ac/index.php) databases to determine pathogenicity.

### Statistical Analysis

SPSS 20.0 software was used for statistical analyses. Categorical data are presented as counts and percentages and were analyzed with Chi-square test and Fisher's exact test and Bonferroni adjustment for multiple analyses as appropriate. Skewed distributions are described with medians and interquartile ranges and were compared by Wilcoxon rank-sum test and Kruskal-Wallis test. Normal distributions were tested by Q-Q analysis, described with means and standard deviations, and compared using Student's *t*-tests. A *P-*value < 0.05 was considered to be statistically significant.

## Results

### Patient Characteristics

We recruited 117 patients using the inclusion and exclusion criteria. Fourteen patients were excluded as follows: four patients did not complete the required information, either due to unsuccessful attempts at the phone interview and/or failure to make contact, three ASD patients had no seizures even though their EEGs were abnormal, six patient had ASD-like symptoms, but the screening scale was negative, and one patient was diagnosed with secondary epilepsy after an upper gastrointestinal hemorrhage. All the patients had an ABC score, eight patients had ADOS scores, three patients had ADI-R scores, and 92 patients met DSM-V criteria. In total, 103 (91.9%) patients were included. Sixty-nine patients suffered epilepsy prior to ASD diagnosis, 28 patients were diagnosed with ASD before epilepsy, and six patients had an unclear relationship between epilepsy and ASD.

**Figure 1:**
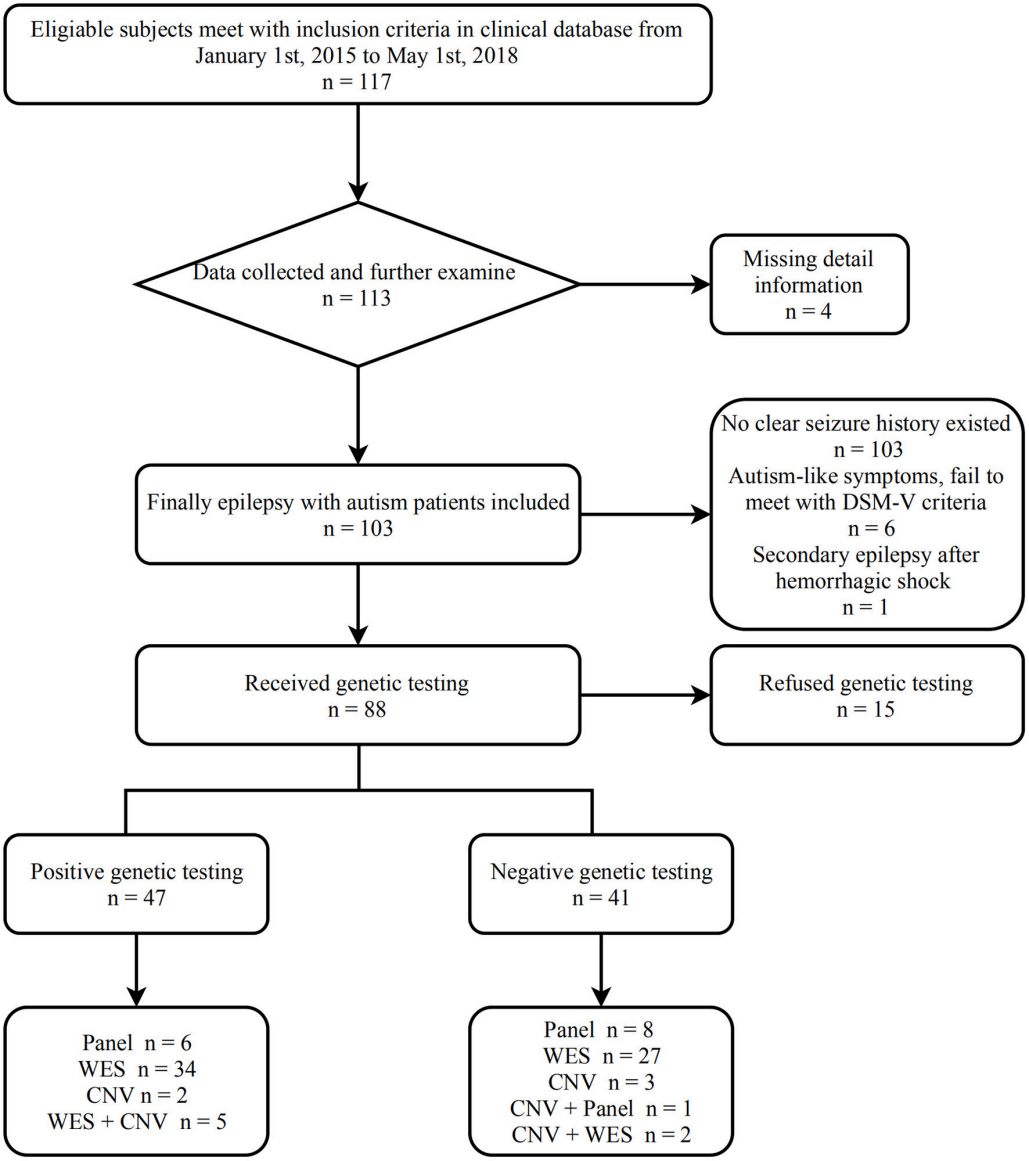
Screening eligibility flow chart.

Figure 1 shows the process from screening to assessment of epilepsy with ASD patients.

We first analyzed the demographic and clinical characteristics of epilepsy with ASD patients (Table 1). The results show that the ratio of male to female patients was 2.4:1, the median age of seizure onset was 12.0 months [interquartile range, 7.0~28.0 months], and the median age of ASD onset was 24.0 months [interquartile range, 24.0~36.0 months]. Overall, 68.9% (71/103) patients were in seizure remission, while the remainder (31.0%, 32/103) were affected by drug-refractory epilepsy. ID (IQ < 70, 81.6%) was high in epilepsy with ASD patients, especially moderate to severe ID (IQ < 50, 58.3%).

**Table 1:** Demographics and clinical features of epilepsy with ASD patients (*n* = 103).

	**Epilepsy with ASD** ***n* = 69 (%)**	**ASD with epilepsy** ***n* = 28 (%)**	**Uncertain** ***n* = 6 (%)**	**Total** ***n* = 103 (%)**
**BASELINE**				
Male	47 (68.4)	21 (75.0)	5 (83.3)	72 (69.9)
Age (years) mean ± SD	5.86 ± 2.90	8.04 ± 3.25	7.82 ± 3.38	6.58 ± 3.16
**CLINICAL FEATURES**				
Age of seizure onset (month) Median (IQR)	10 (6.0, 12.0)	47 (36.0, 60.0)	24 (23.0, 27.0)	12.00 (7.00, 30.00)
**Seizure frequency**				
Frequently attacks	55 (79.7)	23 (82.1)	6 (100%)	84 (81.6)
Infrequently attacks	14 (20.3)	5 (17.9)	0 (0)	19 (18.4)
Status epilepticus	16 (23.2)	7 (25.0)	0 (0)	23 (22.3)
More than two seizure types	28 (40.6)	9 (32.1)	4 (66.7)	41 (39.8)
**Epilepsy type**		
Generalized	39 (56.5)	19 (67.9)	5 (83.3)	63 (61.2)
Focal	17 (24.6)	6 (21.4)	1 (16.7)	24 (23.3)
Combined generalized and focal	13 (18.8)	3 (10.7)	0 (0.0)	16 (15.5)
Duration of seizure (years)	3.35 (2.0, 4.0)	2.9 (1.62, 3.0)	3.5 (1.75, 5.25)	2.00 (2.00, 4.00)
Median (IQR)				
Epilepsy syndrome and epilepsy-associated syndrome	24 (34.8)	8 (28.6)	0 (0)	32 (31.1)
**Other psychological comorbidities**				
Attention deficit hyperactivity disorder	11 (15.9)	5 (17.9)	1 (16.7)	17 (16.5)
Tic disorder	2 (2.9)	0 (1.0)	0 (1.0)	2 (1.9)
Migraine	1 (1.4)	1 (3.8)	0 (1.0)	3 (2.9)
Age of ASD onset (month)	24 (23.25, 36.00)	24 (24.0, 30.0)	24 (24.0, 27.0)	24.00 (24.00, 36.00)
Median (IQR)				
ASD symptoms severe after seizure	13 (18.8)	11 (39.3)	1 (16.7)	25 (24.3)
**ASD severity**				
Mild	12 (17.4)	2 (7.1)	3 (50.0)	17 (16.5)
Moderate	32 (46.4)	14 (50.0)	3 (50.0)	49 (47.6)
Sever	25 (36.2)	12 (42.9)	0 (0.0)	37 (35.9)
**OTHER HISTORY**				
Perinatal abnormalities	17 (24.6)	12 (42.9)	0 (0.0)	29 (28.2)
Family history	11 (15.9)	4 (14.3)	0 (0.0)	15 (14.6)
**Development history**				
Normal	20 (29.0)	6 (21.4)	2 (33.3)	28 (27.2)
Delay	49 (71.0)	22 (78.6)	4 (66.7)	75 (72.8)
Regression	32 (47.1)	11 (40.7)	3 (50.0)	46 (45.5)
Delayed motor development	48 (69.6)	18 (64.3)	4 (66.7)	70 (68.0)
Learning disability	28 (46.7)	14 (51.9)	1 (16.7)	44 (42.7)
Unavailable to assess (<6 years old)	9 (13.0)	1 (3.6)	0 (0.0)	9 (8.7)
**EXAMINATION**				
**EEG**				
No epileptic charge	19 (27.5)	8 (28.6)	1 (16.7)	28 (27.2)
Multifocal charge	18 (26.1)	4 (14.3)	1 (16.7)	23 (22.3)
Focal charge	31 (44.9)	16 (57.1)	4 (66.7)	51 (49.5)
Missing	1 (1.4)	0 (0.0)	0 (0.0)	1 (0.9)
**MRI**				
Normal	28 (40.6)	14 (50.0)	2 (33.3)	44 (42.7)
Abnormal	41 (59.4)	14 (50.0)	4 (66.7)	59 (57.3)
Brain dysplasia	17 (24.6)	6 (21.4)	1 (16.7)	24 (23.3)
**Intelligence quotient**				
IQ > 70	8 (12.3)	3 (12.0)	0 (0.0)	11 (10.7)
50 ≤ IQ ≤ 70	12 (18.5)	1 (4.0)	2 (33.3)	15 (14.6)
IQ < 50	45 (69.2)	21 (84.0)	4 (66.7)	67 (67.0)
Missing	5 (7.2)	3 (10.7)	0 (0.0)	8 (7.8)
**Genetic testing**				
Positive	24 (34.8)	15 (53.6)	2 (33.3)	47 (45.6)
Negative	37 (53.6)	8 (28.6)	2 (33.3)	41 (39.8)
No testing	8 (11.6)	5 (17.9)	2 (33.3)	15 (14.5)
**TREATMENT**				
**AEDs used**				
No AED	2 (2.9)	8 (28.6)	0 (0.0)	10 (9.7)
Monotherapy	29 (42.0)	6 (21.4)	3 (50.0)	38 (36.8)
Polytherapy	38 (55.1)	14 (50.0)	3 (50.0)	55 (53.4)
Seizure-controlled epilepsy	45 (65.2)	20 (71.4)	6 (100.0)	71 (68.9)
Refractory epilepsy	24 (34.8)	8 (28.6)	0 (0.0)	32 (31.1)

*ASD, autism spectrum disorder; MRI, magnetic resonance imagine; EEG, electroencephalogram; IQ, intelligence quotient; AEDs, antiepileptic drugs*.

**Figure 2:**
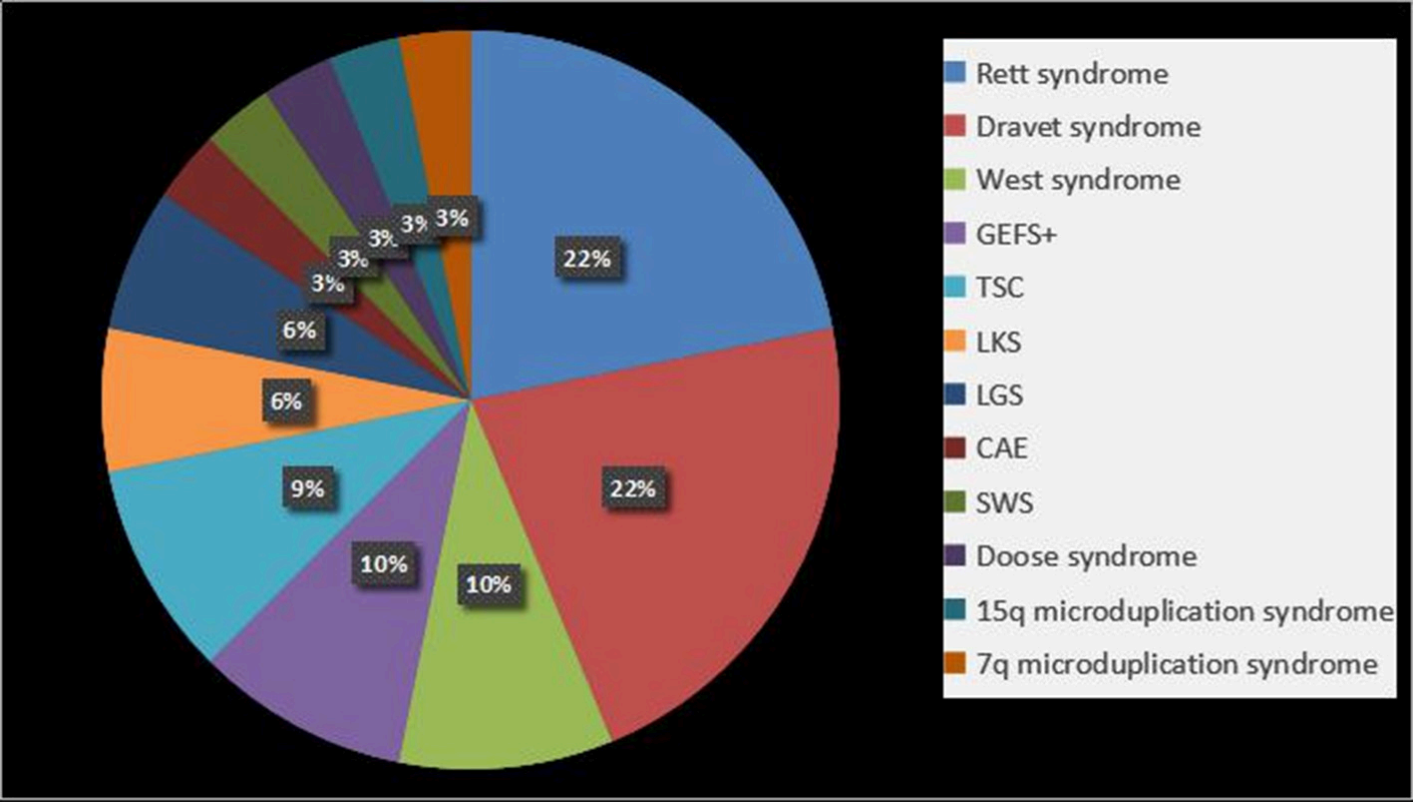
Epilepsy syndrome and epilepsy-associated syndromes in epilepsy with ASD patients. GEFS+, Generalized epilepsy with febrile seizures plus; TSC, tuberous sclerosis complex; LKS, Landau-Kleffner syndrome; LGS, Lennox-Gastaut syndrome; CAE, childhood absence epilepsy; SWS, Sturge-Weber syndrome.

We found that 31.1% patients (32/103) were diagnosed with epilepsy syndrome and epilepsy-associated syndromes, the details of which are shown in Figure 2. Rett syndrome and DS were common in patients (21.8%, 7/32). It should be noted that two of the Rett syndrome patients were diagnosed using clinical criteria, but no variants were identified, and the remainder had clear *MECP2 de novo* gene mutations.

### Genetic Characteristics of Epilepsy With ASD Patients

Eighty (88/103, 85.4%) patients received genetic testing, five patients had CNV analysis, 14 patients had targeted neuromuscular panel results, 61 patients had WES results, seven patients had WES and CNV results, and one patient and CNV and targeted panel results. In all, 53.9% (47/88) were positive for gene mutations and CNVs (Table S1). Gene mutations, involving 25 kinds of genes, were the most common etiological cause and accounted for 83.0% (39/47) of co-occurring epilepsy with ASD patients, with *de novo* mutations accounting for 46.8% (22/47) of patients. Among these genes, *SCN1A* (14.9%, 7/47) was most frequently associated with epilepsy syndromes, such as DS and general febrile seizures (FS). *MECP2* (10.6%, 5/47) was the second most frequent and was associated with Rett syndrome. In our study, of the 5 CNV-negative cases, two patients were negative for WES, two patients could not be contacted, and one patient refused WES.

**Figure 3:**
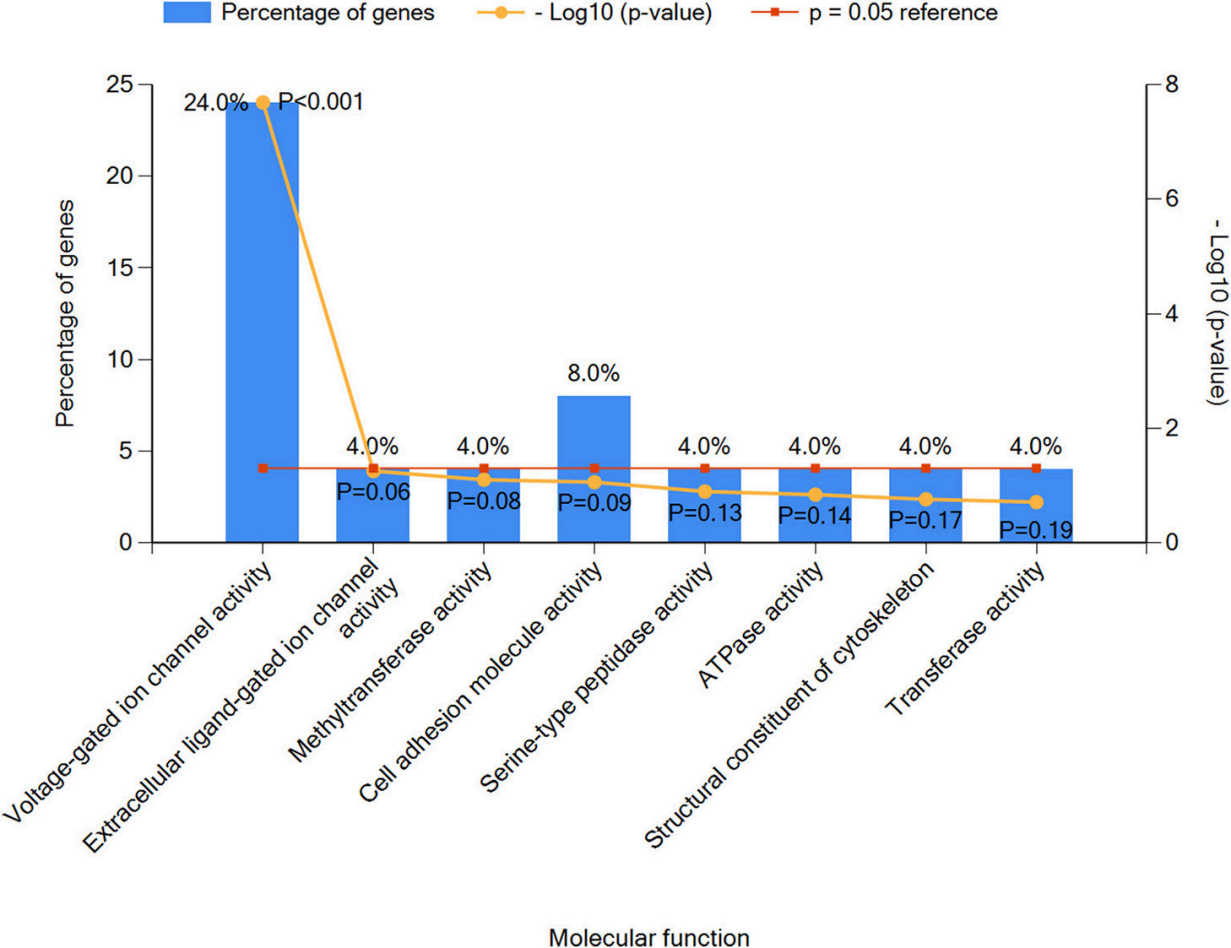
The top eight molecular function classifications of epilepsy with ASD pathogenic genes. Voltage-gated ion channel activity included *CACNA1A, SCN1A, CACNA1H, SCN2A, CACNA1D*, and *KCNQ2*. Extracellular ligand-gated ion channel activity included *GABRG2*. Methyltransferase activity included *EHMT1*. Cell adhesion molecular activity included *CNTNAP2* and *PCDH19*. Serine-type peptidase activity included *TPP1*. ATPase activity included *DYNC1H1*. Structural constituents of the cytoskeleton included *NF2*, and transferase activity included *PIGA*.

Next, we performed an enrichment analysis of positive genes and categorized them into eight subtypes based on molecular functions as seen in Figure 3. Results indicated that voltage-gated ion channel activity genes played an important role in epilepsy with ASD patients, including *SCN1A, SCN2A, CACNA1A, CACNA1H, CACNA1D*, and *KCNQ2* (*P* < 0.05), which were as further classified as sodium ion channel genes, calcium ion channel genes, and potassium ion channel genes (detailed information in Table S2).

**Figure 4:**
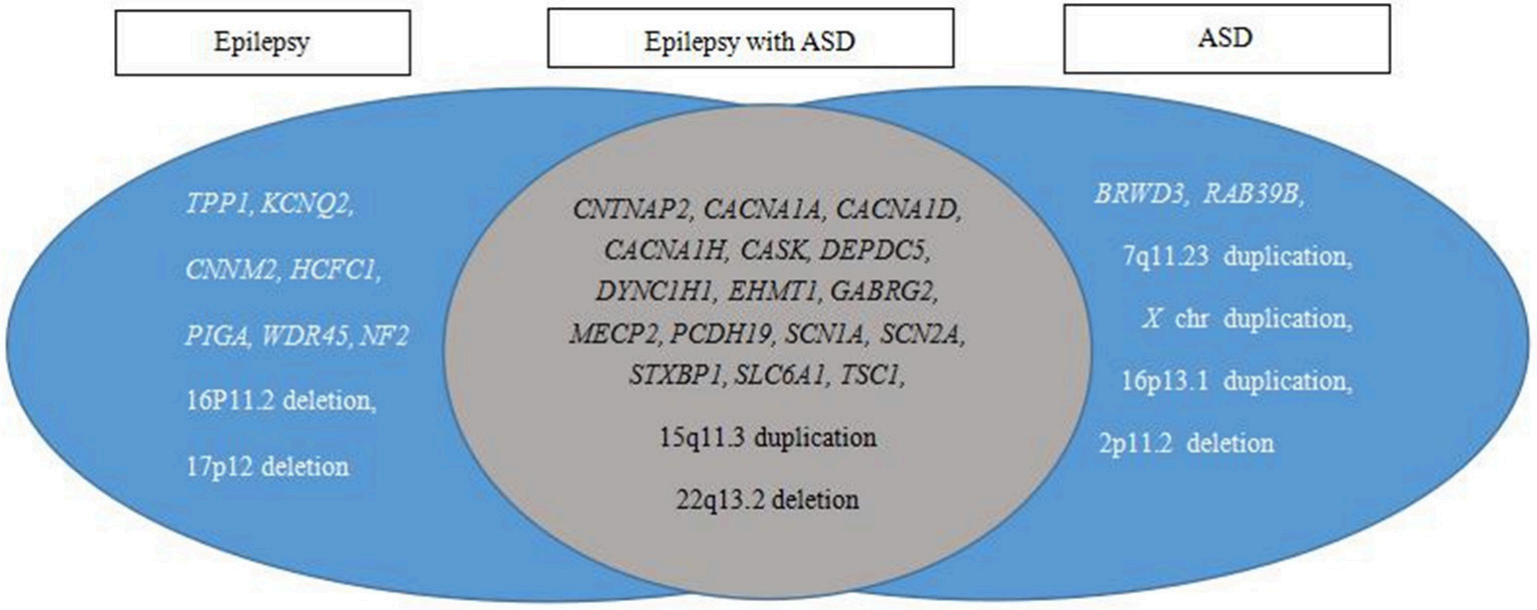
Pathogenic variant category based on reported phenotypes from PubMed, OMIM, and GeneReviews database searches. Epilepsy and ASD have a large overlap in etiology.

The causative variants in 25 genes and eight CNVs were annotated individually for the genotype and phenotype correlations. These variants were classified into three categories, epilepsy, epilepsy with ASD, and ASD, based on published data (Figure 4). The results showed 18 causative variants overlapped in epilepsy and ASD, accounting for 54.5% (18/33) of the examined genes.

### Differences Between the Positive Genetic Test Group and the Negative Genetic Test Group

Comparative analysis of the positive genetic test group and negative genetic test group is shown in Table 2. The results showed that epilepsy with ASD patients who tested positive for genetic abnormalities were prone to higher rates of epilepsy syndrome and epilepsy-associated syndromes (corrected *P* = 0.014). Age of seizure onset was earlier in the positive test group than in the negative test group (corrected *P* = 0.048). The number of AEDs used was higher in the positive test group than in the negative test group (*P* = 0.01). ASD severity, intellectual disability, and other variables were not significantly different (Table 2).

**Table 2:** Comparison of clinical characteristics between the positive genetic test group and the negative genetic test group (*n* = 88).

**Variables**	**Positive genetic test group** **(*n* = 47)**	**Negative genetic test group** **(*n* = 41)**	***P-*value**	*P-*value-corrected^*^
Male (%)	31 (66.0)	28 (68.3)	0.816	0.996
**Age of seizure onset (month)** **Median (IQR)**	**12.0 (6.0, 18.0)**	**12.0 (10.5, 36.0)**	**0.021**	**0.048**
Age of ASD onset (month) Median (IQR)	24.0 (24.0, 36.0)	24.0 (20.0, 36.0)	0.481	0.481
More than 2 seizure types	22 (45.8)	14 (34.1)	0.228	0.323
Epilepsy type			0.423	
Generalized	26 (55.3)	27 (65.9)		
Focal	12 (25.5)	10 (24.4)		
Combined generalized and focal	9 (19.1)	4 (9.8)		
Status epilepsy	10 (21.3)	8 (19.5)	0.838	1.000
**Epilepsy syndrome and epilepsy-associated syndrome**	**22 (46.8)**	**8 (19.5)**	**0.007**	**0.014**
ASD symptoms severe after seizure	22 (46.8)	16 (41.0)	0.591	0.749
MRI abnormal	26 (55.3)	24 (58.5)	0.761	0.930
Cerebral dysplasia in MRI	10 (21.3)	9 (22.0)	0.939	1.000
Multifocal charge in EEG	11 (23.4)	9/40 (22.5)	0.557	
Focal charge in EEG	26 (53.2)	21/40 (51.2)	0.559	
Intelligence quotient			0.707	
IQ > 70	6 (13.0)	4 (10.0)		
50 ≤ IQ ≤ 70	8 (17.4)	5 (12.5)		
IQ < 50	32 (69.6)	31 (77.5)		
Motor delay	35 (74.5)	25 (61.0)	0.175	0.260
ASD severity			0.504	
Mild	5 (10.6)	8 (19.5)		
Moderate	23 (48.9)	18 (43.9)		
Severe	19 (40.4)	15 (36.6)		
**Number of AEDs used mean ± SD**	**2.06 ± 1.09**	**1.46 ± 1.02**	**0.010**	
Refractory epilepsy	19 (40.4)	9 (22.0)	0.063	0.104

*ASD, autism spectrum disorder; MRI, magnetic resonance imagine; EEG, electroencephalogram; IQ, intelligence quotient; AEDs, antiepileptic drugs. bold values mean P < 0.05*.

* *Adjusted for multiple testing using Bonferroni-adjustment*.

### Gene Distribution in the Seizure-Controlled Group and the Drug-Refractory Epilepsy Group

**Figure 5:**
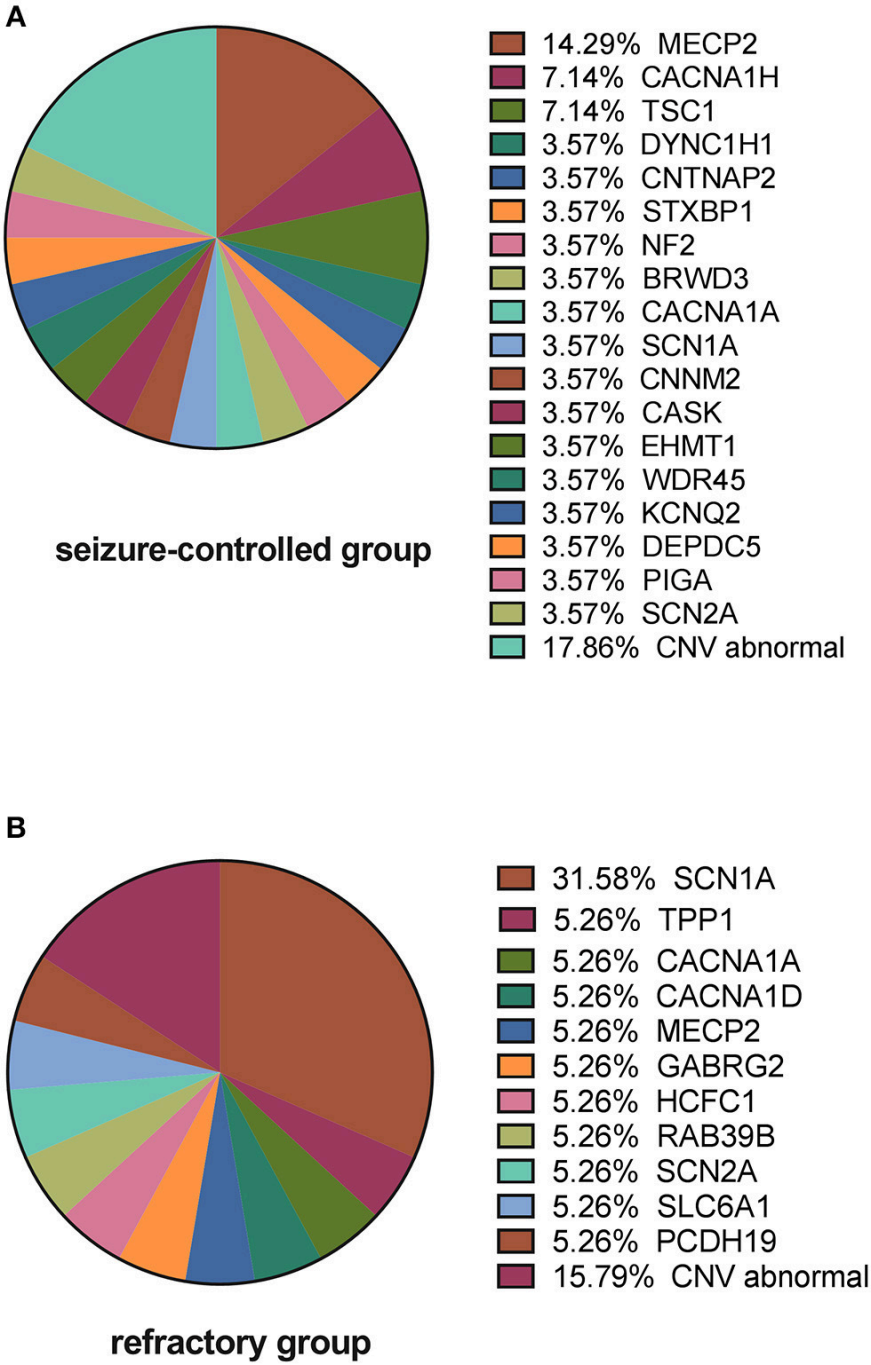
Causative variants in the seizure-controlled group and the refractory group. **(A)** Shows that *MECP2* is the primary gene mutated in the seizure-controlled epilepsy with ASD group, while CNV abnormalities in the seizure-controlled group include 2p 13.2 deletion, 16p 11.2 deletion, 16p 13.11 duplication, 17p 12 deletion, and 22q13.2 deletion. **(B)** Shows that *SCN1A* is the primary gene mutated in the refractory epilepsy with ASD group, while CNV abnormalities in the refractory group include 7q 11.23 duplication, X chromosomal duplication, and 15q 11.3 duplication.

We next analyzed the gene distribution of the two groups and found that *MECP2* mutations were most prominent in the seizure-controlled group (13.7%, 4/29), and *SCN1A* mutations were most prominent in the drug-refractory epilepsy group (31.5%, 6/19) (Figure 5).

## Discussion

Epilepsy and ASD patients have a definite overlap in clinical behavior and etiology, which demonstrates the neural network specificity of the two diseases. Understanding this peculiarity is expected to provide a window for exploring the shared mechanism of these neurodevelopmental disorders. This study provides a genetic spectrum and comprehensive clinical analysis of epilepsy with ASD patients in China. Furthermore, this study provides insight into the possible etiology of these disorders based on genetic molecular function.

Epilepsy with ASD is a group of highly heterogeneous diseases with diverse phenotypes and genotypes and with various comorbidity rates for different epilepsy syndromes. In the current study, Rett syndrome, which is characterized by developmental regression, ASD-like symptoms, and epilepsy and has a higher incidence in females, was the disease most associated with the experimental cohort. Similarly, DS was common in epilepsy with ASD patients due to *SCN1A* gene mutations. The above results were in accordance with previous studies (17, 18). We also found that *SCN1A* mutations were associated with FS, generalized epilepsy with FS plus (GEFS+), and DS. Among these diseases, FS and GEFS+ tend to have benign outcomes, and seizures are easy to control. However, DS is defined as intractable epileptic encephalopathy and usually has poor prognosis.

In the present study, the proportion of patients with drug-refractory epilepsy with ASD was 31.1%, which is close to the reported incidence of drug-refractory epilepsy in the general population with epilepsy (36.7%) (19). However, we are unable to conclude whether seizures are better or worse controlled in epilepsy with ASD without comparative studies involving epilepsy alone. In our study, although no statistical significance was found, drug-refractory epilepsy showed a trend of increased incidence in the positive genetic test group compared with the negative genetic test group, which was likely because drug-refractory epilepsy was more likely have a genetic etiology, such as has been reported for *PCDH19* (20) and *SCN1A* gene mutations (21), which carry a greater risk of drug-refractory epilepsy. The result may be associated with the small sample size used in the study. Epilepsy and ASD often cooccur with varying degrees of developmental delay, intellectual and learning disabilities, and behavioral problems (22). Although we tried to accurately collect both ASD age onset and age of diagnosis (if different), we found it difficult to identify specific ages because ASD symptoms might occur earlier than is normally recognized by either parents or teachers.

In a large population-based cohort study, ASD patients with an IQ <70 had a 5-fold increased risk for epilepsy compared with patients with an IQ > 70 (2). Although most studies showed an increased risk of intellectual disability (23), our study indicated no association between ID severity and drug-refractory epilepsy. Similarly, we found that the degree of ID was not directly associated with increases in seizures. He et al. (24) showed that there was no difference in ID severity between LGS and DS. We therefore speculated that ID might not account for the poor outcomes of epilepsy with ASD patients, as ID, especially moderate to severe ID, was common in both groups.

The current study reinforces the importance of genetic testing for clarifying the etiology and prognosis of epilepsy with ASD patients. From our study, the clinical genetic testing strategy for both epilepsy and ASD were similar to those previously reported (11). First, we used a chromosomal microarray followed by targeted next-generation sequence (NGS) gene panels to determine if possible positive results were evident, after which we used WES. If the parents have another child, we recommend trio-WES, which is conducive to prenatal counseling. NGS sequencing and bioinformatics analysis can simultaneously detect pathogenic variants and copy number variants (>1 Mb). The analysis process includes interpretation of large fragment chromosomal copy number variants. For negative results, due to the limitations of current research methods, it is not possible to completely rule out certain variants, and there may be other types of genetic mutations or non-genetic factors that are difficult to detect and cannot be determined. In fact, to avoid false negative results, children in this study underwent at least one genetic testing method with the informed consent of the guardians, especially when their parents wanted to have a second child.

Although the causal relationship between epilepsy and ASD is not clear, there is a large overlap between the pathogenic genes among the two (52.9%), as seen in Figure 3. Among the genes associated with epileptic encephalopathy in infancy and childhood, 62 genes were associated with epileptic encephalopathy, and as many as 34 genes were candidates for ASD (25). In our study, more than half the patients were positive for genetic testing, and *de novo* genes played an important role in etiology. Through summary analysis of the positive results, we found that ion channel genes accounted for a relatively high proportion, especially those associated with sodium ion channels. The results agree with previous studies on the etiology of epilepsy (26). Specifically, we found the top two most frequently mutated genes to be *SCN1A* and *MECP2*. It has been reported that mutation of *SCN1A* is the most common mutation associated with epilepsy in the clinic (20), especially *de novo* mutations in drug-refractory epilepsy (27). Several reviews have analyzed and summarized gene mutations in epilepsy and ASD and indicated a common overlap between mutated genes and the respective diseases (28). Our findings strengthen evidence for these previous observations. Therefore, we believe there is a strong indication for genetic testing for children with epilepsy and ASD as the results of these tests will likely lead to better diagnosis, more precise treatment, and more accurate prognosis assessment. The results of this study also point to the significance of screening for ASD patients with ion channel genetic mutations related to epilepsy, which may help reduce the rate of missed and delayed diagnosis. In the current study, 15 patients failed to undergo genetic testing for a variety of reasons, including that patients were seizure-free or were easily able to control seizures using AEDs and determined that either it was unnecessary to spend a large sum of money on genetic testing or that WES was too expensive.

The study had several limitations. First, because it was a retrospective study and because a small portion of patients were examined in other hospitals, we failed to collect all the necessary information from patients. Even though best efforts were made, especially for IQ test results, EEG results, and brain MRI scans. Second, as this study was an observational cohort study, we are not able to make statements about causal relationships in epilepsy and ASD. Third, the sample size was too limited to obtain a definitive conclusion, and therefore, a large multicenter cohort study is needed. Finally, the study lacked an ASD specific diagnostic instrument for most ASD patients. All ASD children were recommended for ADOS or ADI-R assessment after clinical diagnosis, but most ASD families refused to participate in because of the long waiting period (which may take about 6 months). They were more eager to go to rehabilitation institutions for language training and behavioral interventions as soon as possible.

However, to the best of our knowledge, this study is the first general, descriptive study of a combination of clinical and genetic characteristics in an epilepsy with ASD population without epilepsy syndrome. Our research enlarged the overlapping gene spectrum by summarizing phenotypes and genotypes. According to the latest review (29), 14 types of genes associated with cooccurring epilepsy and ASD were summarized. This study further expanded the gene profile and added calcium channel genes, such as *CACNA1A* and *CACNA1D*, that had been reported in some cases but not classified yet. In addition, this study was a comprehensive, multidimensional, multiphenotype study that characterized the clinical features of epilepsy with ASD patients.

## Conclusions

In ASD patients with epilepsy, almost half of the patients have a genetic etiology. Among them, *SCN1A* and *MECP2* are responsible for most of the phenotypes associated with these respective disorders. ASD patients with epilepsy are more susceptible to epilepsy syndrome and epilepsy-associated syndrome. There were no differences between intractable seizures, ASD severity, or the degree of ID in etiology-known patients and etiology-unknown patients, and as such, future population-based, multicenter, prospective studies are needed to further confirm the results of the current study. Genetic testing is very important for epilepsy and ASD patients because it may be helpful for pediatricians to determine proper treatment and prognosis evaluation. Finally, in clinical work, it is highly desirable to perform ASD screening for children with epilepsy with ion channel gene mutations.

## Ethics Statement

The study was approved by Children's Hospital of Fudan University ethics committee (NM: (2016) 117). All participants or their parents were made aware of the research and signed written consent forms.

## Author Contributions

SLo collected the data, carried out the analyses, and drafted the initial manuscript. YW designed the study and revised the final manuscript. HZ reviewed and revised the manuscript. SLi, CL, TW, and YM helped collect data and critically reviewed the manuscript. YZ and SZ supplied patient data and revised the manuscript. BW contributed to genetic counseling.

### Conflict of Interest Statement

The authors declare that the research was conducted in the absence of any commercial or financial relationships that could be construed as a potential conflict of interest.

## References

[1] Strzelczyk A Griebel C Lux W Rosenow F Reese JP. The burden of severely drug-refractory epilepsy: a comparative longitudinal evaluation of mortality, morbidity, resource use, and cost using german health insurance data. Front Neurol. (2017) 8:712. 10.3389/fneur.2017.00712 29312132 PMC5743903

[2] Amiet C Gourfinkel-An I Laurent C Carayol J Genin B Leguern E. Epilepsy in simplex autism pedigrees is much lower than the rate in multiplex autism pedigrees. Biol Psychiatry. (2013) 74:e3–4. 10.1016/j.biopsych.2013.01.037 23507000

[3] Viscidi EW Triche EW Pescosolido MF Melean RL Joseph RM Spence SJ. Clinical characteristics of children with autism spectrum disorder and co-occurring epilepsy. PLoS ONE. (2013) 8:e67797. 10.1371/journal.pone.0067797 23861807 PMC3701630

[4] Vignoli A La Briola F Peron A Turner K Vannicola C Saccani M. Autism spectrum disorder in tuberous sclerosis complex: searching for risk markers. Orphanet J Rare Dis. (2015) 10:154. 10.1186/s13023-015-0371-1 26631248 PMC4668631

[5] Berkvens JJ Veugen I Veendrick-Meekes MJ Snoeijen-Schouwenaars FM Schelhaas HJ Willemsen MH. Autism and behavior in adult patients with Dravet syndrome (DS). Epilepsy Behav. (2015) 47:11–6. 10.1016/j.yebeh.2015.04.057 26005841

[6] Li BM Liu XR Yi YH Deng YH Su T Zou X. Autism in Dravet syndrome: prevalence, features, and relationship to the clinical characteristics of epilepsy and mental retardation. Epilepsy Behav. (2011) 21:291–5. 10.1016/j.yebeh.2011.04.060 21620773

[7] Saemundsen E Ludvigsson P Hilmarsdottir I Rafnsson V. Autism spectrum disorders in children with seizures in the first year of life - a population-based study. Epilepsia. (2007) 48:1724–30. 10.1111/j.1528-1167.2007.01150.x 17555525

[8] Matsuo M Maeda T Sasaki K Ishill K Hamasaki Y. Frequent association of autism spectrum disorder in patients with childhood onset epilepsy. Brain Dev. (2010) 32:759–63. 10.1016/j.braindev.2010.05.005 20542395

[9] Lo-Castro A Curatolo P. Epilepsy associated with autism and attention deficit hyperactivity disorder: is there a genetic link? Brain Dev. (2014) 36:185–93. 10.1016/j.braindev.2013.04.013 23726375

[10] Jiang YH Wang Y Xiu X Choy KW Pursley AN Cheung SW. Genetic diagnosis of autism spectrum disorders: the opportunity and challenge in the genomics era. Crit Rev Clin Lab Sci. (2014) 51:249–62. 10.3109/10408363.2014.910747 24878448 PMC5937018

[11] Lee BH Smith T Paciorkowski AR. Autism spectrum disorder and epilepsy: Disorders with a shared biology. Epilepsy Behav. (2015) 47:191–201. 10.1016/j.yebeh.2015.03.017 25900226 PMC4475437

[12] Kwan P Arzimanoglou A Berg AT Brodie MJ Allen Hauser W Mathern G. Definition of drug resistant epilepsy: consensus proposal by the ad hoc Task Force of the ILAE Commission on Therapeutic Strategies. Epilepsia. (2010) 51:1069–77. 10.1111/j.1528-1167.2009.02397.x 19889013

[13] Kokoszka MA McGoldrick PE La Vega-Talbott M Raynes H Palmese CA Wolf SM. Epilepsy surgery in patients with autism. J Neurosurg Pediatr. (2017) 19:196–207. 10.3171/2016.7.PEDS1651 27885946

[14] Panigrahy A Lee V Ceschin R Zuccoli G Beluk N Khalifa O. (2016). Brain dysplasia associated with ciliary dysfunction in infants with congenital heart disease. J Pediatr. 178:141–8. 10.1016/j.jpeds.2016.07.041 27574995 PMC5085835

[15] Barkovich AJ Kuzniecky RI Jackson GD Guerrini R Dobyns WB. Classification system for malformations of cortical development: update 2001. Neurology. (2001) 57:2168–78. 10.1212/wnl.57.12.2168 11785496

[16] Yang L Kong Y Dong X Hu L Lin Y Chen X. Clinical and genetic spectrum of a large cohort of children with epilepsy in China. Genet Med. (2018) 21:564–71. 10.1038/s41436-018-0091-8 29930392 PMC6681813

[17] Chepure AH Somaiya MP Subramanyam AA Kamath RK. (2018). Epileptic encephalopathy and autism: a complex interplay. J Pediatr Neurosci. 13:273–5. 10.4103/jpn.JPN_172_17 30090156 PMC6057174

[18] Lindy AS Stosser MB Butler E Downtain-Pickersgill C Shanmugham A Retterer K. Diagnostic outcomes for genetic testing of 70 genes in 8565 patients with epilepsy and neurodevelopmental disorders. Epilepsia. (2018) 59:1062–71. 10.1111/epi.14074 29655203

[19] Kwan P Brodie MJ. Early identification of refractory epilepsy. N Engl J Med. (2000) 342:314–9. 10.1056/NEJM200002033420503 10660394

[20] van Harssel JJ Weckhuysen S van Kempen MJ Hardies K Verbeek NE de Kovel CG. Clinical and genetic aspects of PCDH19-related epilepsy syndromes and the possible role of PCDH19 mutations in males with autism spectrum disorders. Neurogenetics. (2013) 14:23–34. 10.1007/s10048-013-0353-1 23334464

[21] Liu J Tong L Song S Niu Y Li J Wu X Novel and de novo mutations in pediatric refractory epilepsy. Mol Brain. (2018) 11:48 10.1186/s13041-018-0392-5 30185235 PMC6125990

[22] Salpekar J. Neuropsychiatric effects of epilepsy in developmental disorders. Curr Opin Psychiatry. (2018) 31:109–15. 10.1097/YCO.0000000000000392 29227297

[23] Jokiranta E Sourander A Suominen A Timonen-Soivio L Brown AS Sillanpaa M. Epilepsy among children and adolescents with autism spectrum disorders: a population-based study. J Autism Dev Disord. (2014) 44:2547–57. 10.1007/s10803-014-2126-6 24803367

[24] He N Li BM Li ZX Wang J Liu XR Meng H. Few individuals with Lennox-Gastaut syndrome have autism spectrum disorder: a comparison with Dravet syndrome. J Neurodev Disord. (2018) 10:10. 10.1186/s11689-018-9229-x 29558884 PMC5859706

[25] McTague A Howell KB Cross JH Kurian MA Scheffer IE. The genetic landscape of the epileptic encephalopathies of infancy and childhood. Lancet Neurol. (2016) 15:304–16. 10.1016/S1474-4422(15)00250-1 26597089

[26] Epi4K consortium Epilepsy Phenome/Genome Project Ultra-rare genetic variation in common epilepsies: a case-control sequencing study. Lancet Neurol. (2017) 16:135–43. 10.1016/S1474-4422(16)30359-3 28102150

[27] Mulley JC Scheffer IE Petrou S Dibbens LM Berkovic SF Harkin LA. SCN1A mutations and epilepsy. Hum Mutat. (2005) 25:535–42. 10.1002/humu.20178 15880351

[28] Srivastava S Sahin M. Autism spectrum disorder and epileptic encephalopathy: common causes, many questions. J Neurodev Disord. (2017) 9:23. 10.1186/s11689-017-9202-0 28649286 PMC5481888

[29] Keller R Basta R Salerno L Elia M Autism, epilepsy, and synaptopathies: a not rare association. Neurol Sci. (2017) 38:1353–61. 10.1007/s10072-017-2974-x 28455770

